# The Effect of Pretreatment Cataract Surgery on Selective Laser Trabeculoplasty Outcomes: A One-Year Follow-Up Study

**DOI:** 10.3390/diseases14070247

**Published:** 2026-07-08

**Authors:** Sonja Jandroković, Sania Vidas Pauk, Dina Lešin Gaćina, Lorena Karla Šklebar, Martina Tomić, Tomislav Bulum, Iva Bešlić, Ivan Škegro

**Affiliations:** 1 Department of Ophthalmology, Zagreb University Hospital Center, 10000 Zagreb, Croatia; 2 School of Medicine, University of Zagreb, 10000 Zagreb, Croatia; 3 Vuk Vrhovac University Clinic for Diabetes, Endocrinology and Metabolic Diseases, Merkur University Hospital, 10000 Zagreb, Croatia

**Keywords:** selective laser trabeculoplasty, cataract surgery, pseudophakic eyes, intraocular pressure, glaucoma

## Abstract

Background/Objectives: We aim to determine whether prior cataract surgery affects the intraocular pressure (IOP)-lowering effect of selective laser trabeculoplasty (SLT) in patients with primary open-angle glaucoma (POAG). Methods: This prospective interventional cohort study initially included 92 patients with POAG who were inadequately controlled or intolerant to topical therapy and were treated with SLT. Of these, 84 patients completed all scheduled visits and constituted the final analyzed dataset. Patients were divided into phakic and pseudophakic groups, with cataract surgery performed at least one year before SLT in all pseudophakic eyes. All patients underwent standardized 360-degree SLT by a single specialist. The primary outcome was IOP reduction at one year. Secondary outcomes included percentage IOP reduction and treatment success, defined as ≥20% IOP reduction. Data were analyzed using Statistica^TM^ 14.0.1.25 (TIBCO Software Inc., Palo Alto, CA, USA, USA). Results: Before SLT, the median IOP was 20.5 mmHg in phakic eyes and 21 mmHg in pseudophakic eyes. One year after SLT, median IOP decreased insignificantly in phakic eyes (4.0 mmHg; *p* = 0.262), whereas it decreased significantly in pseudophakic eyes (5.5 mmHg; *p* = 0.004). At one year post-SLT, an IOP reduction of ≥20% was observed in 53.6% of phakic and 72.7% of pseudophakic eyes. Pseudophakic patients were significantly older than phakic patients, as expected, because age-related senile cataract is more common in older individuals, and the groups also differed by gender distribution. This gender imbalance was coincidental and reflected the non-randomized inclusion of POAG patients according to clinical need for SLT rather than predefined matching. In unadjusted analyses, one-year IOP reduction was positively associated with age (*p* = 0.048), pretreatment IOP (*p* < 0.001), and prior cataract surgery (*p* = 0.047). However, after adjusting for age, gender, and baseline IOP in a multivariate sensitivity analysis, prior cataract surgery was not an independent predictor of success; only pretreatment IOP predicted a significant reduction (*p* = 0.031) in the analysis of 84 eyes. Conclusion: Although pseudophakic eyes showed greater unadjusted IOP reduction after SLT over one year, lens status was not an independent predictor after adjustment for potential confounders. Therefore, the observed pseudophakic advantage should be interpreted as a hypothesis-generating association warranting further research into the effect of pseudophakia on SLT response.

## 1. Introduction

Glaucoma, a chronic progressive optic neuropathy characterized by irreversible visual field loss, remains the leading cause of irreversible blindness worldwide, and its global burden is projected to increase substantially over the coming decades [1,2,3,4], with the number of individuals affected by open-angle glaucoma (OAG) projected to increase to 186.6 million by 2060 [1]. Lowering intraocular pressure (IOP) is currently the only proven strategy to slow disease progression [2,3,5]. Although topical hypotensive therapy remains the most used initial approach, its long-term effectiveness may be limited by insufficient IOP control, poor adherence, or ocular surface disease [2,3,6].

Selective laser trabeculoplasty (SLT) has become an established therapeutic option for patients with open-angle glaucoma (OAG) and ocular hypertension (OH) [5,6,7,8]. By selectively targeting pigmented trabecular meshwork cells with a frequency-doubled, Q-switched Nd: YAG laser, SLT enhances aqueous humor outflow through both mechanical and biological mechanisms [6,9,10]. Clinical trials have demonstrated that SLT achieves IOP reductions comparable to those of topical medications, with the LiGHT trial showing that primary SLT provided drop-free IOP control for 74.2% of patients at 36 months and superior cost-effectiveness compared with medications [7,8]. Nevertheless, the IOP response to SLT is not uniform, and variability in treatment outcomes has been attributed to factors such as baseline IOP and angle pigmentation [11,12,13,14,15] as well as prior ocular interventions [16,17,18,19].

One factor with conflicting evidence is the pseudophakic state. Some studies have reported a delayed initial response in pseudophakic eyes at 2 weeks but no overall clinical difference at final follow-up, while others found no significant differences at any time point [16,17]. More recently, prior cataract surgery has also been identified as one of several factors potentially associated with reduced SLT success, together with older age, longer disease duration, and long exposure to topical antiglaucoma medications [19].

Cataract and glaucoma frequently coexist, particularly in elderly patients. Phacoemulsification with intraocular lens implantation has consistently been associated with IOP reduction, with meta-analyses reporting reductions of 3.77 to 5.25 mmHg at 12 months in patients with open-angle glaucoma [20,21]. The proposed mechanisms include deepening of the anterior chamber, widening of the iridocorneal angle, and subsequent improvement in trabecular outflow facility [20,21,22,23,24,25]. The IOP-lowering effect appears more pronounced in eyes with higher preoperative IOP and more crowded anterior segment anatomy, including shallower anterior chambers and narrower iridocorneal angles [20,21].

Given that SLT acts directly at the trabecular meshwork [9,10], whereas cataract surgery modifies anterior segment anatomy and may improve conventional aqueous outflow [20,23,24,25], it is reasonable to hypothesize that structural and biomechanical changes induced by lens extraction could modify the response to subsequent laser trabeculoplasty. While early postoperative changes may transiently affect IOP, evaluating patients who underwent cataract surgery at least one year prior to SLT allows for assessment beyond the immediate surgical effect and short-term inflammatory alterations. However, the long-term influence of prior cataract surgery on SLT efficacy remains unclear in the literature, with existing studies reporting inconsistent results.

Most previous studies directly comparing SLT outcomes by lens status have included mixed OAG and OHT populations, relatively small pseudophakic cohorts, and variable follow-up periods, or have not specifically accounted for the interval between cataract surgery and SLT [16,17,18]. Consequently, whether pseudophakia itself influences the durability of SLT response remains incompletely understood. Moreover, because cataract surgery may exert an independent and sustained effect on IOP, failure to account for the timing of surgery may further confound the interpretation of SLT outcomes. The present study was designed to address this gap by evaluating SLT outcomes in a well-defined cohort of POAG patients, including pseudophakic eyes that had undergone cataract surgery at least one year before SLT, with a 1-year follow-up period.

Accordingly, this study aimed to investigate the influence of lens status on SLT efficacy in patients with POAG who were insufficiently controlled or intolerant to medical therapy by comparing outcomes between phakic and pseudophakic eyes at least 1 year after cataract surgery.

## 2. Patients and Methods

### 2.1. Study Design and Ethics

This prospective interventional clinical cohort study was conducted at the Department of Ophthalmology, Zagreb University Hospital Center, Zagreb, Croatia, in accordance with the Declaration of Helsinki, and was approved by the Hospital’s Ethics Committee (number 02/013 AG, date: 2 March 2023). All participants received oral information about the study purpose and protocol, and signed a written informed consent form.

### 2.2. Participants

A total of 165 eyes (117 phakic, 48 pseudophakic) from 92 patients with POAG who were either insufficiently controlled or intolerant to medical therapy were recruited during the authors’ routine clinical work, treated with SLT according to a standard protocol, and followed for one year. All included eyes had open iridocorneal angles in all quadrants (Shaffer grades III–IV) and mild-to-moderate trabecular meshwork pigmentation (Scheie grades I–II).

Pseudophakic glaucoma patients were those who underwent uncomplicated cataract surgery at least 1 year before SLT, performed by a single cataract surgeon (I.Š.) using an ophthalmic microscope (Lumera 300, Carl Zeiss Meditec AG, Jena, Germany) and a phacoemulsification device (Centurion Vision System, Alcon, Switzerland). The surgery was performed through a 2.75 mm corneal incision at 12 o’clock in the superior position, using phacoemulsification with IOL implantation in the capsular bag. Postoperatively, all patients received the same treatment in addition to glaucoma medication: tobramycin/dexamethasone eye drops three times a day and tobramycin/dexamethasone eye ointment once a day for the first seven days, followed by tobramycin/dexamethasone eye drops three times a day for the next three weeks.

Patients who were not eligible to participate in this study were those with a history of previous selective laser trabeculoplasty or incisional glaucoma surgery, patients who had undergone cataract surgery within the year before the beginning of the study or during the follow-up period, those who had any other type of ocular surgery, or those with other ocular or systemic disease that could affect IOP during the follow-up period.

### 2.3. Scheduled Visits and Ophthalmological Examinations

The first baseline visit was the inclusion visit in the study, done before selective laser trabeculoplasty (referred to as the 0th day), while the four follow-up visits were scheduled for the first, third, and sixth months, and at one year after SLT. Given the potential influence of the daytime on IOP, all visits were scheduled and conducted at 9:00 a.m. (±1 h).

At the first baseline visit, the ophthalmologist collected patients’ demographic and medical history data, and all patients underwent standard ophthalmological examinations, including Snellen visual acuity testing, slit-lamp gonioscopy, examination of the anterior eye segment in the physiological state and after mydriasis with eye drops containing 0.5% tropicamide, and indirect slit-lamp fundoscopy, performed using the Zeiss Slit-lamp (Carl Zeiss Meditec AG, Jena, Germany). IOP was measured with a calibrated Goldman tono-meter on the same slit-lamp. Two measurements were taken, and if they differed by more than 2 mmHg, a third reading was obtained. The average value was used in the analysis. Ophthalmological examinations during the follow-up period were conducted by the second author (S.V.P.), while all clinical findings were independently reviewed and verified by the third author (D.L.G.).

At the four follow-up visits, all patients underwent slit-lamp gonioscopy, anterior segment examination, and Goldman IOP measurement.

Data for the analysis were recorded at baseline and at the four follow-up visits (per protocol). Complete success of SLT was defined as a reduction in IOP of ≥20% after one year without the need for additional glaucoma medication, repeat laser treatment, or glaucoma surgery during the follow-up period. Eyes requiring any of these additional interventions were classified as treatment failures.

### 2.4. Selective Laser Trabeculoplasty

SLT was performed using a frequency-doubled Q-switched Nd: YAG laser (CITO 532, A.R.C. Laser GmbH, Germany), which was calibrated and maintained in accordance with standard protocols. The procedure followed the guidelines set by the European Glaucoma Society [5]. A total of 100 laser spots were applied around the iridocorneal angle, with 25 spots in each quadrant. The power settings ranged from 0.8 to 1.2 mJ, starting at 0.8 mJ. The energy was adjusted until small air bubbles, referred to as “champagne bubbles,” formed at the laser burn site. Then, the power was gradually reduced by 0.1 mJ increments until the visible bubbles disappeared. All participants received brimonidine drops and 250 mg of acetazolamide orally at the end of the procedure. All patients were treated by a single glaucoma specialist (S.J.).

### 2.5. Statistical Analysis

Statistical analyses were performed using Statistica^TM^ 14.0.1.25 (TIBCO Software Inc., Palo Alto, CA, USA). The minimum required sample size was calculated at a 95% confidence level, assuming a 50% expected proportion and a 5% margin of error. After finite population correction for 165 treated eyes, the minimum required sample size was 116 eyes; therefore, the final cohort of 152 eyes from 84 patients was considered adequate. Data normality was assessed using the Kolmogorov–Smirnov and Shapiro–Wilk tests. Continuous and ordinal variables were presented as medians with minimum and maximum values, and categorical variables as numbers and percentages. Between-group comparisons were performed using the Mann–Whitney U test for continuous or ordinal variables and the Chi-square test for categorical variables. Changes in IOP across follow-up visits were assessed using the Friedman ANOVA test, followed by the Wilcoxon signed-rank test for post-hoc comparisons. Correlations between one-year IOP outcomes and baseline characteristics were evaluated using the Spearman Rank-Order correlation test. Variables significantly associated with one-year IOP reduction were entered into multiple regression analysis to identify independent predictors. Statistical significance was set at *p* < 0.05. Analyses were primarily performed at the eye level. However, to account for potential inter-eye correlation among patients with both eyes included, a sensitivity analysis was performed by restricting the dataset to one randomly selected eye per patient (*n* = 84 patients/eyes), and the multiple linear regression model was re-evaluated on this independent subset.

## 3. Results

Of the 165 eyes from 92 patients initially included in the study and treated with SLT, 152 eyes from 84 patients completed all scheduled visits and were followed for 1 year. The main reasons for loss of follow-up were coincidental and related to family or professional obligations. No significant baseline differences were observed between excluded eyes and those included in the final cohort. So, data from these 152 eyes from 84 patients were analyzed and presented. Their baseline demographic characteristics are shown in Table 1. Pseudophakic patients were, as expected, significantly older than phakic patients (77.5 vs. 66 years, *p* < 0.001), which is clinically understandable because senile cataract is age-related and cataract surgery is therefore more frequent in older patients. The two groups also differed significantly by gender (*p* = 0.007): among pseudophakic patients, women predominated (χ^2^ = 14.666, *p* = 0.001), whereas the phakic group had a more balanced gender distribution (χ^2^ = 2.824, *p* = 0.093). This gender imbalance was not related to the study design but occurred coincidentally in this non-randomized cohort of POAG patients included according to the clinical need for SLT. These baseline differences are clinically relevant because age and gender may influence SLT response or reflect variations in disease duration, ocular anatomy, medication, or unmeasured factors. Thus, analyses and interpretations accounted for age and gender as potential confounders in the relationship between lens status and post-SLT IOP reduction.

All included eyes had previously been treated with a median of 3 (2–4) topical glaucoma medications, and 25 (16.4%) patients also received acetazolamide 250 mg orally. In 122 (80%) eyes, the target IOP was not achieved with the aforementioned therapy, and 30 (20%) eyes were intolerant to topical treatment. No significant differences were found in the median of glaucoma medications (Z = 1.557, *p* = 0.119) and the percentage of patients treated with acetazolamide (χ^2^ = 0.459, *p* = 0.458) before SLT between the phakic and pseudophakic groups with different glaucoma types.

Before SLT, the median of IOP in phakic eyes was 20.5 mmHg, and in pseudophakic eyes was 21 mmHg. Although the baseline IOP was slightly higher in the pseudophakic group, the median IOP was lower in the pseudophakic group at every follow-up visit after SLT compared to the phakic group, with a significant difference in the 1st month (*p* = 0.017) and one year after SLT (*p* = 0.020) (Table 2). One year after SLT, the median IOP in phakic eyes decreased insignificantly (20.5 vs. 16.5 mmHg, *p* = 0.262), whereas in pseudophakic eyes the median IOP decreased significantly (21 vs. 15.5 mmHg, *p* = 0.004) compared with baseline values (Table 2).

According to the post-hoc analysis, a significant decrease in IOP in the pseudophakic group was found only between the 0th day (before SLT) and the 1st month after SLT (*p* < 0.001), and a marginal decrease from the 6th month to one year after SLT (*p* = 0.058). No substantial change in the median IOP was observed among other visits (*p* > 0.05). The higher maximum IOP value observed in the pseudophakic group at the sixth-month visit should be interpreted with caution. Because IOP values are presented as medians with minimum–maximum ranges, this maximum value reflects the upper end of the distribution and does not indicate an overall increase in IOP in the group. The median IOP in pseudophakic eyes at six months remained below baseline, suggesting that the elevated maximum value most likely reflects an isolated eye with a suboptimal response or transient IOP fluctuations rather than a general loss of SLT effect.

**Figure 1:**
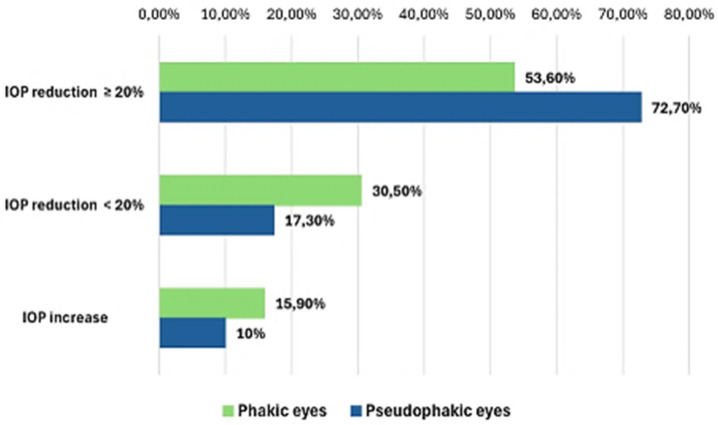
Proportion of phakic and pseudophakic eyes showing treatment success or failure at one year post-SLT.

At one year post-SLT, an IOP reduction of ≥20% without additional glaucoma medication, repeat laser treatment, or glaucoma surgery was observed in 60 phakic eyes (53.6%) and 29 pseudophakic eyes (72.7%) (Figure 1). Eyes that did not achieve ≥20% IOP reduction or required additional glaucoma treatment during follow-up were classified as treatment failures. Thus, in 34 (30.5%) phakic eyes and 7 (17.3%) pseudophakic eyes, the one-year post-SLT IOP reduction was less than 20%, while in 18 (15.9%) phakic and 4 (10%) pseudophakic eyes, an increase in IOP was detected at the end of the study (Figure 1). Among phakic eyes with SLT failures, 11 required additional medication, 20 underwent repeat SLT, and 9 underwent glaucoma surgery; among pseudophakic eyes, 3 required additional medication, and 8 underwent repeat SLT.

The IOP reduction one year after SLT was significantly higher in pseudophakic than phakic eyes (8.0 vs. 4.5 mmHg, *p* = 0.031; 33.3 vs. 23.1%, *p* = 0.037) (Table 3).

The one-year posttreatment IOP reduction was positively related to age (*p* = 0.048), pretreatment IOP (*p* < 0.001), and pretreatment cataract surgery (*p* = 0.047) (Table 4). At the same time, the IOP increase was negatively associated with female gender and positively with male gender (*p* = 0.044) and the presence of cataract (*p* = 0.036). Statistically insignificant associations among other variables are not shown in the table.

The results of the multiple regression analysis for IOP reduction after SLT, expressed as a decrease in mmHg at one-year follow-up, are shown in Table 5. Because the phakic and pseudophakic groups differed significantly in age and gender distributions at baseline, these variables were included in the regression model, along with pretreatment IOP and prior cataract surgery. To account for potential inter-eye correlation, this multivariate model was evaluated as a sensitivity analysis utilizing one randomly selected eye per patient (*n* = 84). After adjustment, only pretreatment IOP remained a significant predictor of IOP reduction (*p* = 0.031), while age, gender, and prior cataract surgery did not reach statistical significance.

## 4. Discussion

In the current prospective interventional cohort study, the authors evaluated whether prior uncomplicated cataract surgery performed at least 1 year before SLT affects the IOP-lowering effect of SLT over a 1-year follow-up period in patients with POAG. The findings demonstrate that pseudophakic eyes achieved significantly greater absolute and percentage reductions in IOP than phakic eyes. Moreover, a higher proportion of pseudophakic eyes reached the predefined success criterion of ≥20% IOP reduction at one year. However, after adjustment for potential confounders in multiple regression analysis, only baseline IOP remained an independent predictor of treatment response.

The IOP-lowering efficacy of SLT observed in the current study is consistent with previously published data reporting a mean reduction of 20% to 30% in patients with OAG and OH (7–8, 12–13). In the present POAG patient cohort, pseudophakic eyes achieved a median reduction of 8.0 mmHg (33.3%), whereas phakic eyes showed a median reduction of 4.5 mmHg (23.1%). Although baseline IOP did not differ significantly between groups, the pseudophakic group exhibited lower median IOP values at most follow-up visits, with a statistically significant difference at 1 month and 1 year.

The superior SLT response observed in pseudophakic eyes in the present study contrasts with findings reported in some previous studies. Shazly et al. found no significant difference in IOP reduction between phakic and pseudophakic eyes at 12 months of follow-up, although they noted a delayed initial response in pseudophakic eyes at 2 weeks [16]. Similarly, Seymenoğlu and Baser reported comparable SLT efficacy regardless of lens status, with no significant differences at any time point during follow-up [17]. These findings are further supported by De Keyser et al., who prospectively compared phakic and pseudophakic eyes and found similar IOP-lowering efficacy between the two groups, with only a tendency toward a faster reduction in pseudophakic eyes [26]. Werner et al. also reported no significant difference in SLT success rates according to lens status, suggesting that pseudophakia does not compromise the therapeutic response to SLT [27]. In contrast, Lindegger et al. observed a weaker early IOP reduction in pseudophakic eyes; however, this difference did not persist during longer follow-up and was not considered clinically relevant [28]. A very recent meta-analysis systematically compared the efficacy of SLT between phakic and pseudophakic eyes in patients with OAG or OH, pooling data from 11 studies and over 1000 eyes. The findings demonstrated no statistically significant difference in IOP reduction or treatment success between the two groups, regardless of follow-up duration [18]. Moreover, the included studies differed in terms of glaucoma subtype, follow-up duration, SLT protocol, and reporting of the interval between cataract surgery and SLT, highlighting the need for more homogeneous study populations and standardized study designs.

However, our findings align with the hypothesis that anatomical changes following cataract surgery may create a more favorable environment for SLT efficacy. Several mechanisms could theoretically explain a more favorable SLT response in pseudophakic eyes. Phacoemulsification induces structural changes in the anterior segment, including deepening of the anterior chamber and widening of the iridocorneal angle [23,24,25]. By reducing anterior segment crowding and improving trabecular meshwork exposure, lens extraction may facilitate more effective laser application during subsequent SLT. In parallel, SLT itself induces biological responses within the trabecular meshwork and conventional outflow pathway, including cellular and inflammatory modulation, as described by Alvarado et al. [10]. However, because the present study found significant age and gender imbalance between groups, and lens status did not remain independently associated with IOP reduction in the adjusted model, these mechanisms should be considered plausible explanations rather than definitive conclusions. This interpretation is also consistent with the recent meta-analysis by Kumbhani et al., which found no statistically significant overall difference in SLT efficacy between phakic and pseudophakic eyes [19].

Hence, when managing patients with coexisting glaucoma and cataract, cataract surgery followed by SLT may be a reasonable treatment sequence in selected patients with visually significant cataract and mild-to-moderate glaucoma. However, the present data do not establish that the pseudophakic state independently improves SLT efficacy. The optimal treatment sequence should therefore be individualized, considering disease severity, visual function, baseline IOP, ocular anatomy, medication burden, and patient preferences [20,21,22,23,24,25].

Importantly, cataract surgery was performed at least one year prior to SLT in all pseudophakic patients in the present study, thereby minimizing the influence of early postoperative inflammation or transient IOP fluctuations. This design strengthens the assumption that the observed differences are related to long-term structural effects rather than short-term surgical consequences. Previous studies [16,17,18,26,27,28] comparing SLT outcomes by lens status did not consistently report or standardize the interval between cataract surgery and SLT, potentially introducing confounding variables related to postoperative inflammation or incomplete anatomical stabilization [23,24,25,29]. Unlike most studies included in the recent meta-analysis [18], the present study specifically evaluated pseudophakic eyes that had undergone cataract surgery at least one year before SLT, thereby minimizing the influence of transient postoperative anatomical and inflammatory changes.

Nevertheless, when age, gender, baseline IOP, and prior cataract surgery were included in a multivariate model, only pretreatment IOP remained a significant independent predictor of IOP reduction. This finding aligns with numerous previous studies demonstrating that higher baseline IOP is the strongest predictor of SLT success [11,12,14,15]. The apparent advantage observed in pseudophakic eyes may therefore be mediated by baseline IOP dynamics, age-related ocular characteristics, gender distribution, disease duration, medication exposure, or other unmeasured variables rather than lens status alone.

Despite the lack of an independent effect of lens status in the multivariable model, the consistently greater IOP reduction observed in pseudophakic eyes remains clinically noteworthy. From a clinical perspective, the present findings contribute to a better understanding of factors associated with variability in SLT response among patients with POAG. Given the frequent coexistence of glaucoma and cataract in the aging population, improved knowledge of the relationship between lens status and SLT outcomes may help clinicians better interpret treatment responses, counsel patients regarding expected outcomes, and individualize management strategies. While our results do not establish a causal effect of prior cataract surgery on SLT efficacy, they support further investigation into the potential role of lens status in shaping treatment outcomes and should be considered when designing future studies evaluating predictors of SLT success.

The present study has several notable strengths. Its prospective design, standardized SLT protocol performed by a single glaucoma specialist, and uniform cataract surgical technique performed by a single experienced surgeon ensured a high degree of procedural consistency. Furthermore, the study evaluated SLT outcomes in a well-defined cohort of POAG patients, including pseudophakic eyes that had undergone cataract surgery at least one year before SLT, thereby minimizing the potential influence of transient postoperative effects on treatment response. The consistent timing of IOP measurements further reduced the impact of diurnal fluctuations, while the one-year follow-up period provided a meaningful assessment of SLT’s sustained efficacy.

However, several limitations must be acknowledged. First, the non-randomized design introduces potential selection bias. Second, the pseudophakic group was smaller than the phakic group, which potentially reduces statistical power. Third, although the phakic group showed a median IOP reduction at one year post-SLT, this did not reach statistical significance in the repeated-measures analysis, likely due to the relatively wide range of IOP values in the phakic group at baseline and follow-up, the inter-individual variability in SLT response, and the initial inclusion of both eyes from some patients in the primary dataset. Importantly, current recommendations for ophthalmic research advise that only one eye per patient be included in statistical analyses when appropriate methods for paired-eye data are not used [30]. To address potential within-patient inter-eye correlation and ensure eye-level observations were fully independent, we performed a robust sensitivity analysis using exactly one randomly selected eye per patient (*n* = 84). This analysis completely eliminated any potential unit-of-analysis bias and confirmed that our primary clinical conclusions remained entirely unchanged, reinforcing the precision of our estimated effects and *p*-values. Fourth, although the present study was designed as a prospective interventional cohort study to evaluate the overall efficacy of SLT in phakic and pseudophakic patients with POAG, several clinically relevant covariates that may influence SLT response were not included in the analysis. These include glaucoma severity, visual field indices, cup-to-disc ratio, central corneal thickness, detailed medication classes, and duration of topical therapy. The relatively limited cohort size, particularly within the pseudophakic group, precluded further stratification according to these variables, as this would have resulted in small subgroups and reduced statistical power. Importantly, most previous studies comparing SLT outcomes between phakic and pseudophakic eyes also did not systematically incorporate these variables into adjusted predictive models, although some studies reported selected baseline characteristics [16,17,18,26,27,28]. Therefore, residual confounding cannot be excluded, and the observed association between pseudophakia and greater unadjusted IOP reduction should be interpreted cautiously rather than as evidence of an independent effect of lens status. Furthermore, although the one-year follow-up period allowed assessment of medium-term treatment efficacy, longer-term studies are needed to determine the durability of the observed differences between phakic and pseudophakic eyes. Finally, the use of a single experienced cataract surgeon and a single glaucoma specialist performing all SLT procedures may be viewed as both a strength and a limitation of the study. While this approach ensured procedural consistency and minimized inter-operator variability, it may also have limited the generalizability of the findings and introduced operator-related bias. Therefore, confirmation of our results in larger multicenter studies involving multiple surgeons and treatment providers is warranted.

## 5. Conclusions

In this prospective interventional cohort study, pseudophakic eyes achieved greater unadjusted IOP reduction following SLT than phakic eyes, and a higher proportion of pseudophakic eyes met the predefined ≥20% IOP reduction success criterion at one year. However, the pseudophakic patients were significantly older, as expected, because senile cataract is age-related and cataract surgery is therefore more common in older individuals. The observed gender imbalance was coincidental and reflected the non-randomized inclusion of POAG patients according to clinical need for SLT rather than predefined matching. After adjustment for age, gender, and baseline IOP in a robust sensitivity analysis utilizing one eye per patient, lens status was not an independent predictor of IOP reduction; therefore, the apparent advantage observed in pseudophakic eyes may reflect confounding by demographic or other unmeasured clinical factors. Cataract surgery followed by SLT may remain a reasonable individualized treatment sequence in selected patients with coexisting glaucoma and visually significant cataract, but future randomized or adequately adjusted prospective studies with larger sample sizes, detailed anterior segment imaging, and alternative paired-eye statistical models are warranted to further clarify whether lens status independently influences SLT outcomes.

## Figures and Tables

**Table 1:** Baseline demographic characteristics of glaucoma patients included in the study.

	POAG Patients (*n* = 84)	Phakic Group (*n* = 60)	Pseudophakic Group (*n* = 24)	Z ^a^ χ^2 b^	*p*-Value
Age (years) *	72 (37–85)	66 (37–75)	77.5 (69–85)	−5.268 ^a^	<0.001
Gender (m/f) **	37/47	32/28	5/19	7.347 ^b^	0.007

Legend: Values are * median (min-max) and ** numbers. ^a^ and Z indicate the Mann-Whitney U test, and ^b^ and χ^2^ the Chi-square test.

**Table 2:** Intraocular pressure on the 0th day (before SLT) and during the follow-up period in the phakic and pseudophakic groups.

	IOP (mmHg)						
	0th day before SLT	1st month after SLT	3rd month after SLT	6th month after SLT	One year after SLT	χ^2 b^	*p*-Value
Phakic group	20.5 (11–33)	19 (13–28)	17.5 (12–28)	17.2 (10–33)	16.5 (10–30)	3.254	0.262
Pseudophakic group	21 (12–29)	18 (11–26)	17 (11–28)	17 (13–34)	15.5 (8–23)	9.563	0.004
Z ^a^	−0.231	2.382	1.044	0.358	2.274		
*p*-Value	0.817	0.017	0.296	0.720	0.020		

Legend: Values are median (min-max). ^a^ and Z indicate the Mann-Whitney U test, ^b^ and χ^2^ the Friedman ANOVA test, IOP intraocular pressure, and SLT selective laser trabeculoplasty.

**Table 3:** Intraocular pressure reduction one year after SLT in the phakic and pseudophakic groups.

	Phakic Group	Pseudophakic Group	Z	*p*-Value
IOP reduction after SLT (mmHg)	4.5 (0–12)	8.0 (0–13)	−2.949	0.031
IOP reduction after SLT (%)	23.1 (0–50)	33.3 (0–52)	−2.288	0.037

Legend: Values are median (min-max). Z indicates the Mann–Whitney U test; IOP, intraocular pressure; and SLT, selective laser trabeculoplasty.

**Table 4:** Correlation between the one-year posttreatment IOP reduction and increase, and baseline demographic and clinical characteristics in all glaucoma patients included in the study.

		Spearman R	t(N-2)	*p*-Value
The one-year posttreatment IOP reduction	Age	0. 399	2.087	0.048
	Pretreatment IOP	0.593	5.259	<0.001
	Pretreatment cataract surgery	0. 276	2.027	0.047
The one-year posttreatment IOP increase	Gender (male/female)	−0.888	−3.354	0.044
	Presence of cataract	0.563	2.361	0.036

Legend: Spearman R indicates the Spearman Rank-Order correlation test, and IOP indicates the intraocular pressure.

**Table 5:** Results of multiple regression analysis for the IOP reduction one year after SLT.

	*b*	Std. Err. of *b*	t(19)	*p*-Value
Age	0.303	0.271	1.119	0.276
Gender (male/female)	−9.034	5.324	−1.697	0.105
Pretreatment IOP	2.339	1.008	2.321	0.031
Pretreatment cataract surgery	1.281	6.539	0.196	0.847

Legend: *b* indicates the unstandardized coefficient and Std. Err. of *b* indicates the standard error of the coefficient in multiple regression analysis, IOP is intraocular pressure, and SLT is selective laser trabeculoplasty.

## Data Availability

The authors confirm that the data supporting the findings of this study are available within the article and from the corresponding author upon request.
